# Effect of infusion of M&G solution for protection of renal tissue in Wistar rats subjected to programmed ischemia-reperfusion

**DOI:** 10.1590/1677-5449.190010

**Published:** 2020-06-06

**Authors:** Leandro Pablos Rossetti, Larissa Bastos Eloy da Costa, Ana Terezinha Guillaumon

**Affiliations:** 1 Universidade Estadual de Campinas – UNICAMP, Departamento de Cirurgia Vascular, Campinas, SP, Brasil.; 2 Universidade Estadual de Campinas – UNICAMP, Departamento de Anatomia Patológica, Campinas, SP, Brasil.

**Keywords:** ischemia-reperfusion, renal failure, preservation solution

## Abstract

**Background:**

Renal ischemia-reperfusion (I/R) is directly associated with acute renal failure and can occur in conditions such as infarction caused by embolization or thrombosis, septicemia, and kidney transplantation. The process is complex, involving innate and adaptive immune responses, presence of cellular infiltrate, and production and release of cytokines and chemokines. It also triggers cell responses and release of reactive oxygen species, in addition to causing apoptosis and, in some cases, cell necrosis. Against this background, evaluation of renal tissue protection mechanisms is essential.

**Objectives:**

The objective of this study was to test the M&G solution, developed in prior research, evaluating its capacity to protect the kidneys using morphometric analysis and by assaying the presence and expression of inflammatory cytokines (TNF-alpha, VEGF, HIF, and IL-8).

**Methods:**

Eighteen Wistar rats were divided into three groups: Sham (S), Control (C), and Experimental (E). The S group underwent the surgical operation, but without arterial clamping. In group C, the aorta was clamped above and below the left renal artery, without infusion of the preservation solution. In group E, in addition to clamping, the aorta was punctured and M&G solution was infused continuously for 20 minutes at 15^o^ C. Morphological analysis and immunohistochemical assessment of markers were then conducted.

**Results:**

Morphological differences were identified in group S compared with groups C and E. Analysis of markers revealed reduced intensity of expression of TNF and of VEGF in group E. There were no differences in HIF or IL-8 between groups.

**Conclusions:**

The M&G solution was associated with a reduction in presence and expression of TNF-alpha and a trend to reduced VEGF.

## INTRODUCTION

The kidneys are the organs responsible for homeostasis of the body, regulating tubular reabsorption of water, ions, glucose, and nutrients and removing metabolic products by glomerular filtration. The process of renal ischemia-reperfusion (I/R) is directly associated with acute renal failure and can occur in conditions such as infarction caused by embolization or thrombosis, septicemia, and kidney transplantation. It is characterized by restriction of the blood flow available to the organ, followed by reestablishment of the blood supply. During this process, many compensatory and harmful mechanisms are triggered. These changes are associated with high rates of morbidity and mortality.^1^^,^^2^

The changes provoked by the lack of blood and, consequently, of oxygen supply to cells, produce an inflammatory cascade, resulting in reduced production of adenosine triphosphate (ATP) by mitochondrial oxidative phosphorylation and increased glycolysis, which is the anaerobic process for releasing energy.^3^ This involves complex vascular and cellular changes, triggering structural and functional changes in renal tissues. Proximal tubule cells are more sensitive to ATP privation than the cells in Henle’s loop or distal tubules, because of the high metabolic rate needed for ion transport and the limited capacity to work in an anaerobic state.^4^^,^^5^

Cytokines are molecules that have the capacity to regulate growth, death, and differentiation and function of cells. Thus, metabolic activity of renal tissues can be evaluated through inflammatory mediators, identifying the intensity of reactions and, therefore, the proportions of the changes present in tissues as a result of the ischemia-reperfusion process.^6^

Against this background, it is important to evaluate the activity of preservation solutions that are capable of reducing the degree of injury caused by this process. There are several solutions that can reduce tissue damage, such as Collins Solution, University of Wisconsin Solution, and Custodiol, combined or not with hypothermia.^7^^,^^8^ In an attempt to improve on these, M&G solution was developed with extracellular characteristics, and therefore a lower potassium (K^+^) content, aiming to reduce injury. This solution was developed at the Vascular Research and Microprocedure Laboratory at the Universidade Estadual de Campinas (UNICAMP), in Brazil.^9^

**Figure 1:**
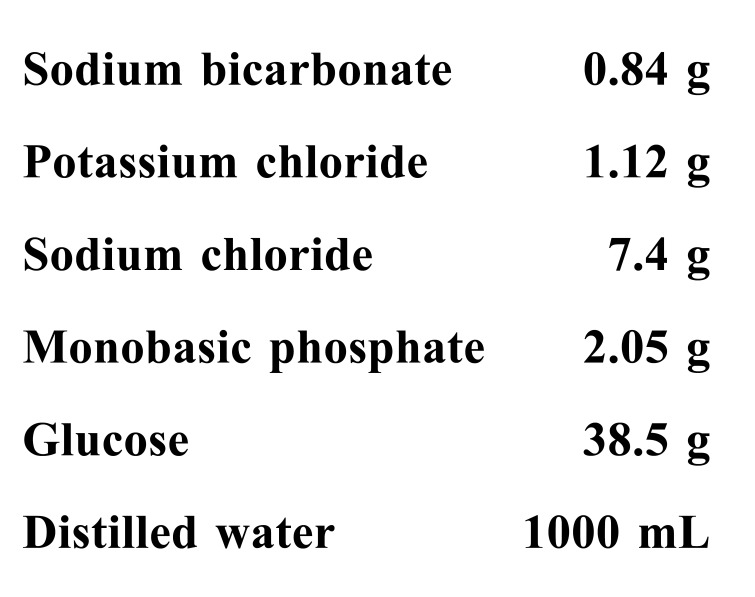
Composition of M&G solution.

The objectives were to evaluate the possible protective effects of M&G solution (Figure 1) at low temperatures (15 °C) in the renal tissues of Wistar rats subjected to programmed ischemia-reperfusion, by analyzing the following cytokines: tumor necrosis factor alpha (TNF-alpha), hypoxia-induced factor (HIF), vascular endothelial growth factor (VEGF), and interleukin 8 (IL-8).

## MATERIALS AND METHODS

### Experiment

M&G solution was developed to have extracellular characteristics, with a higher quantity of Na^+^ and a lower quantity of K^+^, as electrolytes. Phosphate buffer was used, with glucose as the membrane-impermeable agent, achieving a pH of 7.74 (Figure 1).

In order to evaluate the protective function of the solution, 18 male Wistar rats bred under conventional conditions were obtained from the university’s Central Animal House after approval by the Animal Usage Ethics Committee (CEUA - no. 4077-1). The animals were divided into three groups: Sham (S), Control (C), and Experimental (E). They were anesthetized with intraperitoneal ketamine/xylazine, not exceeding the maximum dose of 80/10 milligrams per kilogram respectively. The experiment was conducted under controlled temperature conditions (23 °C). After anesthesia, the rats underwent abdominal shaving followed by antisepsis with alcoholic 2% iodine solution.

Surgery initiated with a midline laparotomy and then the animal was randomized into one of the groups. In group S, structures were dissected without clamping and without infusion of the solution. In group C, the aorta was clamped above and below the left renal artery, without infusion of the solution. In group E, clamping was performed, followed by infusion of 1 milliliter of M&G solution at 15 °C, continually for 20 minutes, via puncture of the aorta. After removal of the catheter, it was necessary to suture the aorta with 10.0 nylon monofilament. The abdominal wall was then closed with 4.0 nylon monofilament.

The rats were kept under observation for 7 days, during which time their diet was reintroduced and they were offered oral analgesic. They were kept in an artificial 12-hour light/dark cycle until euthanasia in a carbon dioxide chamber.

### Analysis of renal tissues

The left kidneys were harvested from the animals in each group and processed to produce histological slides. The examiner was unaware of which group each animal belonged to and slides were analyzed in random order. The tissues were first analyzed for morphology using Hematoxylin-Eosin staining. The objective was to detect morphological changes caused by I/R, observing changes such as pyknotic nuclei, karyolysis, acidophilia, and loss of the tubule framework. This analysis was performed using images captured with a Nikon 995 digital camera fitted to the microscope (Axio Lab.A1, Zeiss). Histomorphometric analysis was conducted with the aid of IMAGEJ® software.

Next, slides were stained with immunohistochemical reactions with the following reagents: tumor necrosis factor alpha (TNF-alpha), hypoxia-induced factor (HIF), vascular endothelial growth factor (VEGF), and interleukin 8 (IL-8). The same software was used for these analyses. The initial analysis was to determine expression of markers, deriving an index of positivity for the fields evaluated. This was then converted to an 8-bit grayscale. After these steps, semiautomatic segmentation was conducted using the Threshold tool, correcting marking of interest and reducing background marking. The quantity of pixels in each image could then be determined, providing a numerical value corresponding to the intensity of marking.^10^

The Kruskal-Wallis test was used to compare inflammatory markers and intensity of reactions between the three groups of rats (S, C, and E), because the variables were not normally distributed and the groups were small. The significance level adopted for the statistical tests was 5%, i.e., p < 0.05. Statistical analyses were conducted using SAS for Windows, version 9.2, (SAS Institute Inc., 2002-2008, Cary, NC, United States).

## RESULTS

### Morphological assessment

**Figure 2:**
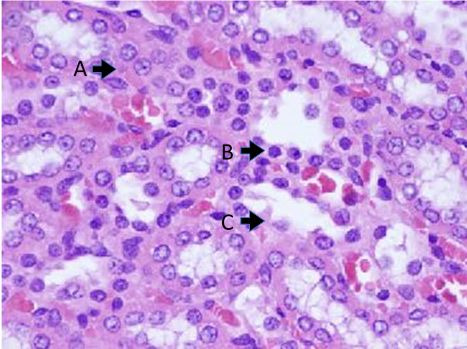
Presence of morphological changes providing evidence of the acute tubular necrosis process in group C. (A) acidophilia; (B) pyknotic nucleus; (C) loss of tubular framework.

Optical microscopy analysis of slides stained with H&E from groups S, C, and E detected structural changes, primarily in the region of the renal cortex, where there is significant metabolic activity of tubules (Figures 1 and 2). This analysis identified statistically significant differences between group S and groups C and E (p = 0.006). No differences were detected between groups C and E.

### Immunohistochemical analysis

**Figure 3:**
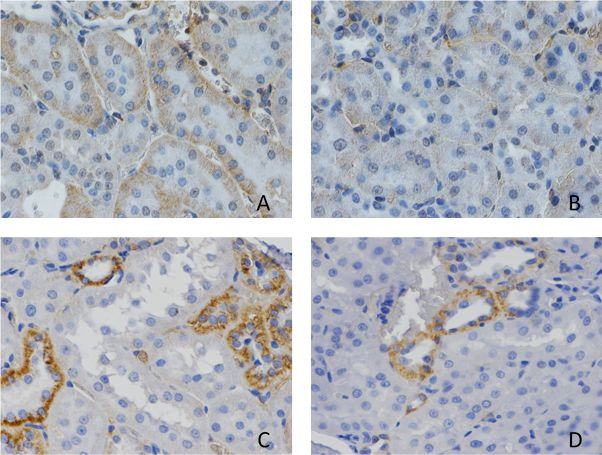
Immunohistochemical study of renal tissues. (A) control group marked with tumor necrosis factor (TNF); (B) Experimental group marked with TNF (there were differences in intensity of TNF expression, which was lower with the preservation solution); (C) Control group marked with vascular endothelial growth factor (VEGF); (D) Experimental group marked with VEGF (a trend was observed for reduced expression with use of the preservation solution).

The immunohistochemical analysis identified presence of staining and identified antibodies that are primarily located in cytoplasm (Figures 2 and 3). Table 1, below, shows comparisons of the results for inflammatory markers and the intensities of the reactions of markers in each of the three groups of rats.

**Table 1:** Comparison of inflammatory markers and reaction intensity in three groups of rats: Sham (S), Control (C), and Experimental (E).

**GROUP**	**Variable**	**N**	**Mean**	**SD**	**Min**	**Q1**	**Median**	**Q3**	**Max**	**p** ^*^
S	TNF	6	11.05	12.19	0.00	0.00	9.75	13.70	33.10	p = 0.002 → S≠E, S≠C
	IL-8	6	59.78	29.05	22.80	34.20	64.45	85.00	87.80	p = 0.268
	VEGF	6	29.82	13.19	12.40	18.90	30.15	39.50	47.80	p = 0.038 → S≠C
	HIF	6	0.00	0.00	0.00	0.00	0.00	0.00	0.00	p = 0.006 → S≠E, S≠C
										
	IntensTNF	6	52.74	7.28	41.19	46.91	55.09	58.40	59.73	p < 0.001 → S≠E, S≠C, E≠C
	IntensIL-8	6	52.20	7.84	41.92	47.95	51.02	56.70	64.60	p = 0.003 → S≠E, S≠C
	IntensVEGF	6	55.96	13.65	32.10	50.63	57.83	67.67	69.73	p = 0.751
	IntensHIF	6	47.49	7.01	40.23	40.84	46.70	51.63	58.85	p = 0.128
										
C	TNF	6	94.55	9.58	76.50	90.80	100.00	100.00	100.00	
	IL-8	6	82.32	23.01	37.70	84.50	85.85	100.00	100.00	
	VEGF	6	47.25	10.66	37.10	39.60	42.90	60.10	60.90	
	HIF	6	15.97	8.90	0.90	10.70	19.05	22.50	23.60	
										
	IntensTNF	6	78.55	5.83	68.90	74.83	80.32	82.39	84.56	
	IntensIL-8	6	76.50	5.18	69.48	72.18	77.28	80.24	82.56	
	IntensVEGF	6	54.85	7.93	42.81	48.18	56.57	61.51	63.48	
	IntensHIF	6	45.06	5.32	40.67	40.71	42.92	50.70	52.44	
										
E	TNF	6	81.98	16.14	53.40	74.70	87.45	88.90	100.00	
	IL-8	6	82.63	14.93	67.00	70.00	79.40	100.00	100.00	
	VEGF	6	33.90	6.94	21.70	29.60	32.95	35.40	50.80	
	HIF	6	9.67	9.41	0.00	0.00	2.97	11.68	24.00	
										
	IntensTNF	6	67.46	4.12	60.37	65.35	68.29	71.08	71.36	
	IntensIL-8	6	73.28	7.05	65.50	65.89	72.73	80.76	82.06	
	IntensVEGF	6	57.12	9.76	44.20	46.87	59.72	64.45	67.78	
	IntensHIF	6	53.87	6.96	48.60	49.71	51.08	55.51	67.23	

SD = standard deviation; HIF = hypoxia-induced factor; IL-8 = interleukin 8; TNF = tumor necrosis factor; VEGF = vascular endothelial growth factor; IntensHIF = HIF Reaction intensity; IntensIL-8 = IL-8 Reaction intensity; IntensTNF = TNF Reaction intensity; IntensVEGF = VEGF Reaction intensity; N = number of rats in each group; Q1 = 25th percentile; Q3 = 75th percentile; Min = minimum value; Max = maximum value.

* p values according to Kruskal-Wallis test to compare variables between the three groups.

The statistical tests showed that there were differences between the three groups for TNF-alpha, with p < 0.05. There were no differences between the three groups when total values for IL-8 were compared, but there was a difference in the intensity of the reaction for this cytokine between group S and groups C and E.

For VEGF, there was a difference between group S and group C, with a higher result for the second. There were no differences between groups S and E or between groups C and E, but there was a trend for reduced expression of VEGF in group E compared with group C. There were no differences in intensity of reaction to expression of VEGF.

HIF was not identified in group S. Therefore, when compared with groups C and E, there were significant increases in expression of the marker when subjected to warm or cold ischemia with protection.

## DISCUSSION

The mechanisms of renal ischemia-reperfusion are complex and involve several pathways such as hypoxia, release of reactive oxygen species, build up of neutrophils, and release of oxygen free radicals and lytic enzymes. The morphofunctional changes that result from this process are related to the duration of ischemia and the tissue’s capacity to tolerate anaerobiosis.^3^^,^^5^

The analyses of renal tissue conducted primarily focus on changes observed in the cortex, where there is a concentration of proximal tubules, which have considerable metabolic activity for hydroelectrolytic regulation. Efforts to improve techniques and reduce injury have been concentrated on the I/R process. Hypothermia has been widely employed with this objective, because it slows cellular metabolism and reduces oxidative stress and inflammation of tissues.^10^ In addition to hypothermia, there are also preservation solutions that can be used with the objective of improving environments with intracellular or extracellular characteristics.^8^

The morphometric analysis was able to identify significant differences between group S (not subjected to I/R) and groups C and E. The injuries provoked in these renal segments provide evidence of acute tubular necrosis using optical microscopy criteria: pyknotic nuclei, karyorrhexis, and/or cell membrane rupture. These changes have been widely confirmed in the literature and are evidence of the injuries caused by the I/R process. In this model, which is considered acute because of the short duration of ischemia (20 min), using the M&G protective solution in cold ischemia was not capable of preventing structural changes to the renal tissues, when compared with group C. This duration of ischemia is reaffirmed, with evidence of relatively discrete injuries in renal tissues after warm ischemia.^11^

The ideal characteristics of a preservation solution are linked with reduced cellular activity in the renal parenchyma, lower antigenicity, nontoxic osmotic agents, and energetic substrates that incorporate peroxides, which maintain the cell membranes more stable. In addition to these factors, the composition, pressure, and duration of perfusion are extremely important for conservation of the renal tissues.^12^

Production of TNF-alpha is related to bursts produced by reactive oxygen species, caused by I/R. The effects of this molecule on the kidneys are related to reduction of glomerular blood flow and filtration rate and induction of synthesis of other proinflammatory mediators, such as IL-1. Glomerular permeability is also increased, provoking fibrin deposition and stimulating cellular infiltration by activation of adhesion molecules, such as ICAM-1 and selectin, promoting apoptosis.^13^^,^^14^

When immunohistochemical results were assessed, in the form of counts of cells positive for the TNF-alpha marker, it was observed that there was a difference between group S and groups C and E. No difference was observed between groups C and E, but when the intensity of the reaction was evaluated by analysis of pixels, the intensity was greater in group C than in group E. This is evidence that the inflammatory process had lower intensity in the group with M&G preservation solution. Studies evaluating use of allopurinol in renal I/R also found evidence of lower TNF-alpha levels, similar to what was observed with M&G solution.^15^

VEGF is released during the ischemia-reperfusion process. This factor has a function in neovascularization, with endothelial proliferation, migration, and remodeling.^16^ This process has been confirmed by Hao,^17^ who assessed expression using messenger RNA tests for VEGF production, which was elevated after I/R. In the experiment conducted, this elevation of VEGF expression was identified in the comparison between groups S and C. There was no statistically significant difference when group E was compared with the other groups. There is therefore a tendency for the inflammatory process to be reduced and for lower expression of angiogenesis when the preservation solution is used. Under normal conditions, the endothelium does not exhibit exacerbated mitotic activity, but in response to the stimuli caused by ischemia and increased production of HIF, stimulating VEGF production, angiogenesis occurs and permeability of blood vessels is increased, regulating vasculogenesis.^18^

In view of the known importance of the process of the response to ischemia, HIF was analyzed, since it has a protein regulation function, as part of tissue adaptation. Inhibition of HIF during I/R indicates intensification of the harmful response, whereas accumulation is protective.^19^ When the three groups were compared, there were no differences in HIF expression or reaction intensity. In previous evaluations of M&G solution, infused during the I/R process in limbs subjected to varying durations of ischemia (180 min), the solution exhibited a certain degree of protection of perfused tissues, comparing longer periods of exposure to ischemia when HIF was analyzed and an absence of differences between groups when VEGF was analyzed.^9^

The principal function of IL-8 is its capacity to activate the leukocytic activation process, making injuries provoked during I/R more likely. It normally has low expression in the body, but, in response to minimal stimulation it tends to increase during this process.^20^ No differences were found between the groups in the results for expression, but differences were observed in intensity of staining, confirming the low expression in periods without I/R aggression and higher expression in periods of metabolic stress.

The limitations of this study are linked to the low number of organisms in each group, to the 20-minute ischemia period, and to the lack of a comparative analysis with other preservation solutions. There is a need to validate the renal protection process in further studies.

## CONCLUSIONS

The process of renal ischemia-reperfusion is a complex chain of reactions that can trigger molecular and structural changes. In this context, a protective effect of M&G solution at 15 °C was identified in comparison with the effect of ischemia without infusion of the preservation solution. There was evidence of reductions in the presence and expression of TNF-alpha, in addition to a trend for reduced VEGF. No differences were detected in the analyses of IL-8 or HIF.
