# Supplementary material for: Effect of infusion of M&G solution for protection of renal tissue in Wistar rats subjected to programmed ischemia-reperfusion
Source: J Vasc Bras. 2020 Jun 6;19:e20190010. doi: 10.1590/1677-5449.190010 (PMC8202179; doi:10.1590/1677-5449.190010)
Supplement: Translation

# Translation: Efeito da infusão da solução M&G na proteção do tecido renal de ratos Wistar submetidos a isquemia e reperfusão programada

**Authors:** Leandro Pablos Rossetti, Larissa Bastos Eloy da Costa, Ana Terezinha Guillaumon

**Affiliations:** 1 Universidade Estadual de Campinas – UNICAMP, Departamento de Cirurgia Vascular, Campinas, SP, Brasil.; 2 Universidade Estadual de Campinas – UNICAMP, Departamento de Anatomia Patológica, Campinas, SP, Brasil.

**Keywords:** isquemia e reperfusão, insuficiência renal, solução preservadora

## Abstract

**Contexto:**

A isquemia e reperfusão (I/R) renal está envolvida diretamente com insuficiência renal aguda, ocorrendo em casos como infarto por embolização ou trombose, quadros de septicemia e transplante renal. Esse processo é complexo, envolvendo respostas imunes inatas e adaptativas, presença de infiltrado celular, produção e liberação de citocinas e quimiocinas. Também desencadeia respostas celulares e liberação de espécies reativas de oxigênio, além de resultar em apoptose e, em alguns casos, necrose celular. Nesse contexto, é imprescindível a avaliação dos mecanismos de proteção ao tecido renal.

**Objetivos:**

O objetivo foi testar a solução desenvolvida M&G, avaliando sua capacidade protetora no rim por meio de análise morfométrica e presença e expressão de citocinas inflamatórias (TNF-alfa, VEGF, HIF e IL-8).

**Métodos:**

Foram selecionados 18 ratos Wistar, divididos em três grupos: Sham (S), Controle (C) e Estudo (E). O grupo S foi submetido ao processo cirúrgico sem o clampeamento arterial. No grupo C, foi clampeada a aorta acima e abaixo da artéria renal esquerda, sem a infusão de solução preservadora. No grupo E, além do clampeamento, realizou-se a punção da aorta e a infusão contínua da solução M&G por 20 minutos a 15 °C. Realizou-se a avaliação morfológica e imuno-histoquímica com os marcadores.

**Resultados:**

Identificaram-se diferenças morfológicas entre o grupo S comparado aos grupos C e E. Na análise dos marcadores, houve redução na intensidade de expressão do TNF e na expressão do VEGF no grupo E. Não houve diferenças com HIF e IL-8 entre os grupos.

**Conclusões:**

A solução M&G apresentou redução da presença e expressão de TNF-alfa e tendência de redução do VEGF.

## INTRODUÇÃO

O rim é o órgão responsável pela homeostasia do organismo, regulando a reabsorção tubular de água, íons, glicose e nutrientes e removendo produtos metabólicos por meio da filtração glomerular. O processo de isquemia e reperfusão (I/R) renal está envolvido diretamente com a insuficiência renal aguda, ocorrendo em casos como infartos por embolização ou trombose, quadros de septicemia e transplantes renais. Caracteriza-se por restrição no fluxo sanguíneo disponível ao órgão e um posterior restabelecimento do fornecimento de sangue. Durante o processo, ocorre o disparo de diversos mecanismos compensatórios e lesivos. Essas alterações estão associadas a elevadas taxas de morbimortalidade^1^^,^^2^.

As alterações provocadas pela falta de sangue e, consequentemente, de fornecimento de oxigênio para as células produzem uma cascata de inflamação, resultando na diminuição da produção de adenosina trifosfato (ATP) por meio de fosforilação oxidativa mitocondrial e aumento da glicólise, o que caracteriza o processo anaeróbio de obtenção de energia^3^. Isso envolve complexas alterações vasculares e celulares, desencadeando mudanças estruturais e funcionais para o tecido renal. As células tubulares proximais são mais sensíveis à privação de ATP do que as células da alça de Henle e dos túbulos distais, por causa da alta taxa de metabolismo requerido no transporte de íons e limitada capacidade de trabalho em meio anaeróbio^4^^,^^5^.

As citocinas são moléculas capazes de regular o crescimento, a morte, a diferenciação e a função das células. Assim, a atividade metabólica dos tecidos renais pode ser avaliada por mediadores inflamatórios, identificando a intensidade das reações e, portanto, as proporções das alterações encontradas no tecido em decorrência do processo de isquemia e reperfusão^6^.

Neste contexto, é importante a avaliação das soluções preservadoras capazes de diminuir o grau de lesão provocada por esse processo. Há diversas soluções que resultam em menores danos teciduais, como a Solução de Collins, *University of Wisconsin* (UW), Custodiol, associada ou não a hipotermia^7^^,^^8^. Na tentativa de aprimoramento, foi desenvolvida a solução M&G, com características extracelular [ou seja, menor quantidade de potássio (K^+^)], buscando ser menos lesiva. A solução foi desenvolvida no laboratório de Microprocedimento e Pesquisa Vascular da Universidade Estadual de Campinas (UNICAMP)^9^.

Os objetivos foram avaliar os possíveis efeitos protetores da solução M&G (Figura 1) à baixa temperatura (15 °C) no tecido renal de ratos Wistar submetidos a isquemia e reperfusão programada, através da análise das seguintes citocinas: fator de necrose tumoral alfa (TNF-alfa), fator induzido pela hipóxia (HIF), fator endotelial de crescimento vascular (VEGF) e interleucina 8 (IL-8).

**Figura 1:** Composição da solução M&G.

## MATERIAL E MÉTODOS

### Experimento

A solução M&G foi desenvolvida com características extracelulares, contendo maior quantidade de Na^+^ e menor quantidade de K^+^, como eletrólitos. Utilizou-se do fosfato com tampão e da glicose como impermeabilizante, apresentando um pH de 7,74 (Figura 1).

Para a avaliação da função protetora da solução, foram selecionados 18 ratos machos Wistar, criados sob condições convencionais, fornecidos pelo Biotério Central da Universidade por meio de aprovação no Comitê de Ética na Utilização de Animais (CEUA – nº. 4077-1). Eles foram divididos em três grupos: Sham (S), Controle (C) e Estudo (E). Os animais foram submetidos a anestesia com quetamina/xilazina por via intraperitoneal, não ultrapassando a dose máxima de 80/10 miligramas por quilograma, respectivamente. O experimento foi realizado em condições de temperatura ambiente controlada (23 °C). Seguiu-se com a tricotomia abdominal do rato e antissepsia com álcool iodado a 2%.

A cirurgia teve início com laparotomia mediana e, então, foi sorteado a qual grupo o animal participaria. No grupo S, realizou-se dissecção das estruturas sem o clampeamento e sem a infusão da solução. No grupo C, realizou-se o clampeamento da aorta acima e abaixo da artéria renal esquerda, sem a infusão da solução. No grupo E, realizou-se o clampeamento e a infusão de 1 mililitro da solução M&G a 15 °C, continuamente, por um período de 20 minutos, através de punção da aorta. Após a retirada da cateterização, foi necessária a sutura da aorta com mononylon 10.0. Procedeu-se o fechamento da parede abdominal com mononylon 4.0.

Os ratos ficaram em observação por 7 dias, durante os quais foi reintroduzida a dieta e oferecido analgésico via oral, e eles foram mantidos em um ciclo artificial claro/escuro de 12 horas até a eutanásia em câmara de gás carbônico.

### Avaliação do tecido renal

Foram coletados os rins esquerdos dos referidos grupos, após eutanásia, e, então, foi feito o processamento com a produção de lâminas histológicas. O avaliador não tinha conhecimento prévio do grupo ao qual estava analisando, examinando de forma aleatorizada as lâminas do estudo. Os tecidos inicialmente foram avaliados em morfologia com a coloração de Hematoxilina e Eosina. O objetivo foi verificar as alterações morfológicas produzidas pela I/R, observando mudanças como núcleos picnóticos, cariólise, acidofilia e perda do arcabouço tubular. Para isso, foram coletadas as imagens com câmera digital Nikon, modelo 995, acoplada ao microscópio (Axio Lab.A1, Zeiss). A análise histomorfométrica foi realizada com ajuda do *software* IMAGEJ®.

Na sequência, foram corados com reações imuno-histoquímicas os seguintes reagentes: fator de necrose tumoral alfa (TNF-alfa), fator induzido pela hipóxia (HIF), fator endotelial de crescimento vascular (VEGF) e interleucina 8 (IL-8). Utilizou-se o mesmo *software* nessa avaliação. A análise inicialmente foi da expressão dos marcadores, sinalizando uma razão de positividade nos campos avaliados. Realizou-se a conversão para a escala de cinza de 8 bits. Após esses passos, foi realizada uma segmentação semiautomática através da ferramenta Threshold, corrigindo a marcação de interesse e diminuindo a marcação de fundo. Assim, pode-se avaliar a quantidade de *pixels* de cada imagem, fornecendo um valor numérico correspondente à intensidade da marcação^10^.

Para a comparação dos marcadores inflamatórios e intensidade de reação entre os três grupos de ratos (S, C e E), foi utilizado o teste de Kruskal-Wallis devido à ausência de distribuição Normal das variáveis e do tamanho reduzido dos grupos. O nível de significância adotado para os testes estatísticos foi de 5%, ou seja, p < 0,05. Para a análise estatística, foi utilizado o programa computacional SAS para Windows (SAS Institute Inc., 2002-2008, Cary, NC, EUA), versão 9.2.

## RESULTADOS

### Avaliação morfológica

A análise em microscopia óptica das lâminas coradas com H&E dos grupos S, C e E evidenciou alterações estruturais principalmente na região do córtex renal, pela ampla atividade metabólica dos túbulos (Figuras 1 e 2). Nessa análise, identificou-se diferenças estatisticamente significantes comparando o grupo S com os grupos C e E (p = 0,006). Não se evidenciou diferenças entre os grupos C e E.

**Figura 2:** Presença de alterações morfológicas evidenciando processo de necrose tubular aguda no grupo C. (A) acidofilia; (B) núcleo picnótico; (C) perda do arcabouço tubular.

### Avaliação imuno-histoquímica

Na avaliação imuno-histoquímica, buscou-se as presenças de coloração e identificação dos anticorpos que se localizam principalmente no citoplasma (Figuras 2 e 3). A Tabela 1, a seguir, apresenta as comparações dos marcadores inflamatórios e das intensidades de reação dos marcadores entre os três grupos de ratos.

**Figura 3:** Estudo da imuno-histoquímica do tecido renal. (A) grupo Controle marcado com fator de necrose tumoral (TNF); (B) grupo Estudo marcado com TNF (houve diferenças na intensidade de expressão do TNF, menor com a solução preservadora); (C) grupo Controle marcado com fator endotelial de crescimento vascular (VEGF); (D) grupo Estudo marcado com VEGF (evidenciou-se tendência de diminuição da expressão com o uso da solução preservadora).

**Tabela 1:** Comparação dos marcadores inflamatórios e da intensidade de reação entre os três grupos de ratos: Sham (S), Controle (C) e Estudo (E).

**GRUPO**	**Variável**	**N**	**Média**	**DP**	**Mín**	**Q1**	**Mediana**	**Q3**	**Máx**	Valor de p*
S	TNF	6	11,05	12,19	0,00	0,00	9,75	13,70	33,10	p = 0,002 → S≠E, S≠C
	IL-8	6	59,78	29,05	22,80	34,20	64,45	85,00	87,80	p = 0,268
	VEGF	6	29,82	13,19	12,40	18,90	30,15	39,50	47,80	p = 0,038 → S≠C
	HIF	6	0,00	0,00	0,00	0,00	0,00	0,00	0,00	p = 0,006 → S≠E, S≠C
										
	IntensTNF	6	52,74	7,28	41,19	46,91	55,09	58,40	59,73	p < 0,001 → S≠E, S≠C, E≠C
	IntensIL-8	6	52,20	7,84	41,92	47,95	51,02	56,70	64,60	p = 0,003 → S≠E, S≠C
	IntensVEGF	6	55,96	13,65	32,10	50,63	57,83	67,67	69,73	p = 0,751
	IntensHIF	6	47,49	7,01	40,23	40,84	46,70	51,63	58,85	p = 0,128
										
C	TNF	6	94,55	9,58	76,50	90,80	100,00	100,00	100,00	
	IL-8	6	82,32	23,01	37,70	84,50	85,85	100,00	100,00	
	VEGF	6	47,25	10,66	37,10	39,60	42,90	60,10	60,90	
	HIF	6	15,97	8,90	0,90	10,70	19,05	22,50	23,60	
										
	IntensTNF	6	78,55	5,83	68,90	74,83	80,32	82,39	84,56	
	IntensIL-8	6	76,50	5,18	69,48	72,18	77,28	80,24	82,56	
	IntensVEGF	6	54,85	7,93	42,81	48,18	56,57	61,51	63,48	
	IntensHIF	6	45,06	5,32	40,67	40,71	42,92	50,70	52,44	
										
E	TNF	6	81,98	16,14	53,40	74,70	87,45	88,90	100,00	
	IL-8	6	82,63	14,93	67,00	70,00	79,40	100,00	100,00	
	VEGF	6	33,90	6,94	21,70	29,60	32,95	35,40	50,80	
	HIF	6	9,67	9,41	0,00	0,00	2,97	11,68	24,00	
										
	IntensTNF	6	67,46	4,12	60,37	65,35	68,29	71,08	71,36	
	IntensIL-8	6	73,28	7,05	65,50	65,89	72,73	80,76	82,06	
	IntensVEGF	6	57,12	9,76	44,20	46,87	59,72	64,45	67,78	
	IntensHIF	6	53,87	6,96	48,60	49,71	51,08	55,51	67,23	

DP = desvio padrão; HIF = fator induzido pela hipóxia; IL-8 = interleucina 8; TNF = fator de necrose tumoral; VEGF = fator endotelial de crescimento vascular; IntensHIF = Intensidade do HIF; IntensIL-8 = Intensidade da IL-8; IntensTNF = Intensidade do TNF; IntensVEGF = Intensidade do VEGF; N = número de ratos de cada grupo; Q1 = percentil 25; Q3 = percentil 75; Min = valor mínimo; Máx = valor máximo.

* Valor de p referente ao teste de Kruskal-Wallis para comparação das variáveis entre os três grupos.

Verificou-se, pelos resultados estatísticos, que para o TNF-alfa houve diferenças entre os três grupos com p < 0,05. Não houve diferença entre os três grupos quando comparados pela IL-8 nos valores totais, mas houve diferença no grupo S em comparação aos grupos C e E na intensidade da citocina.

O VEGF evidenciou diferença entre o grupo S e o grupo C, estando mais presente no último. Não houve diferenças entre os grupos S e E e os grupos C e E, mas houve tendência de diminuição da expressão dos valores de VEGF no grupo E quando confrontado com o grupo C. Nas intensidades de reação à expressão do VEGF, não houve diferença.

O HIF não foi identificado no grupo S. Portanto, quando comparado aos grupos C e E, houve aumento significativo na expressão do marcador quando submetido a isquemia quente ou fria com proteção.

## DISCUSSÃO

Os mecanismos de isquemia e reperfusão renal são complexos e envolvem diversas vias como hipóxia, liberação de espécies reativas de oxigênio, acúmulo de neutrófilos, liberação de radicais livres de oxigênio e enzimas líticas. As alterações morfofuncionais resultantes desse processo estão relacionadas ao tempo de isquemia e à capacidade do tecido de tolerar a anaerobiose^3^^,^^5^.

As análises realizadas do tecido renal concentraram-se principalmente nas alterações do córtex por lá encontrarem-se os túbulos proximais, que apresentam ampla atividade metabólica para a regulação hidroeletrolítica. No processo de I/R, foram concentrados esforços para melhorar as técnicas e diminuir as lesões provocadas. A hipotermia, por sua ação de lentificação do metabolismo celular e redução do estresse oxidativo e da inflamação dos tecidos, tem sido amplamente utilizada com esse fim^10^. Ademais, existem soluções preservadoras que também têm por objetivo melhorar os ambientes com características intra ou extracelulares^8^.

Na análise morfométrica, foi possível identificar diferenças significativas entre o grupo S (não submetido a I/R) e os grupos C e E. Nas lesões provocadas nesse segmento renal, ficam evidentes alterações que representam necrose tubular aguda com critérios de microscopia óptica: núcleo picnótico, cariorréxis e/ou ruptura da membrana celular. Essas alterações são amplamente corroboradas pela literatura e evidenciaram a presença de lesões decorrentes do processo de I/R. A isquemia fria associada à solução protetora M&G, nesse quadro que é considerado agudo pelo tempo curto de isquemia (20 min), não foi capaz de impedir alterações estruturais no tecido renal quando comparado ao grupo C. Esse tempo de isquemia é reafirmado evidenciando injúrias relativamente discretas no tecido renal com a isquemia quente^11^.

As características ideais para a solução preservadora estão vinculadas a menor atividade celular do parênquima renal, menor antigenicidade, agentes osmóticos não tóxicos, substratos energéticos e incorporadores de peróxidos, os quais mantêm mais estável a membrana celular. Além disso, a composição, a pressão e o tempo de perfusão são de extrema importância para a conservação do tecido renal^12^.

A produção do TNF-alfa está relacionada a disparos produzidos pelas espécies reativas de oxigênio, decorrentes da I/R. Os efeitos dessa molécula sobre o rim estão relacionados à diminuição do fluxo de sangue glomerular e da taxa de filtração, além de induzir a síntese de outros mediadores pró-inflamatórios, como IL-1. Também aumenta a permeabilidade glomerular, provocando o depósito de fibrina e estimulando a infiltração celular por ativação de moléculas de adesão, como ICAM-1 e selectina, e promove a apoptose^13^^,^^14^.

Quando se avaliou a imuno-histoquímica e, portanto, a contagem de células positivas para a o marcador TNF-alfa, identificou-se alterações entre o grupo S e os grupos C e E. Não se observou diferenças entre os grupos C e E, mas, quando se avaliou a intensidade de reação obtida pela análise dos *pixels*, a intensidade foi maior no grupo C comparado ao grupo E. Isso evidencia uma menor intensidade do processo inflamatório com a solução preservadora M&G. Estudos avaliando o uso de alopurinol na I/R renal também evidenciam diminuição dos níveis de TNF-alfa, semelhante ao observado com a solução M&G^15^.

Durante o processo de isquemia e reperfusão, ocorre a liberação de VEGF que tem função de neovascularização, com proliferação, migração e remodelamento endotelial^16^. Esse processo é reafirmado por Hao^17^, que avaliou a expressão por testes de RNA mensageiro da produção de VEGF, elevada após I/R. No experimento realizado, essa elevação da expressão do VEGF foi identificada comparando os grupos S e C. Na avaliação do grupo E com os demais grupos, não houve diferença estatisticamente significante. Assim, há uma tendência de diminuição do processo inflamatório e uma menor expressão da angiogênese com o uso da solução preservadora. Normalmente, o endotélio não tem atividade mitótica exacerbada, mas, em decorrência do estímulo produzido pela isquemia e pela maior produção de HIF, estimulando a produção de VEGF, decorre a angiogênese e o aumento da permeabilidade dos vasos sanguíneos, regulando a vasculogênese^18^.

Sabendo da importância do processo de resposta para a isquemia, foi avaliado o HIF que tem função de regulação proteica, como adaptação tecidual. A inibição do HIF durante a I/R mostra a intensificação da resposta deletéria, enquanto o acúmulo é protetor^19^. Não houve diferenças na expressão e intensidade da reação do HIF quando comparados os três grupos. Em avaliações anteriores da solução M&G na infusão durante processo de I/R de membros com variações de tempo de isquemia (180 min), a solução apresentou certa proteção aos tecidos perfundidos comparando períodos maiores de exposição a isquemia na avaliação do HIF e ausência de diferenças entre os grupos quanto ao VEGF^9^.

A IL-8 tem como principal função a capacidade de ativar o processo de ativação leucocitária, favorecendo as lesões provocadas durante a I/R. Normalmente, tem baixa expressão no organismo, mas, ao mínimo estímulo, tende a se elevar durante esse processo^20^. Nos resultados, não foram encontradas diferenças na expressão entre os grupos, mas evidenciaram-se diferenças na intensidade de coloração, corroborando a expressão baixa em períodos sem a agressão da I/R e mais elevadas em períodos de estresse metabólico.

As limitações do trabalho são vinculadas ao número reduzido de organismos em cada grupo, ao tempo de 20 minutos de isquemia e à não análise comparativa com outras soluções preservadoras. O processo de proteção renal necessita de mais estudos para ser validado.

## CONCLUSÃO

O processo de isquemia e reperfusão renal é uma complexa cadeia de reações que podem desencadear alterações moleculares e estruturais. Nesse contexto, identificou-se o efeito protetor da solução M&G a 15 °C comparado com o efeito da isquemia sem infusão da solução preservadora. Verificou-se uma redução da presença e expressão do TNF-alfa, além de tendência de diminuição do VEGF. Não se verificou diferenças nas avaliações de IL-8 e HIF.
